# Supplementary figures and images for: Under Stress: Searching for Genes Involved in the Response of Abies pinsapo Boiss to Climate Change
Source: Int J Mol Sci. 2024 Apr 28;25(9):4820. doi: 10.3390/ijms25094820 (PMC11084517; doi:10.3390/ijms25094820)

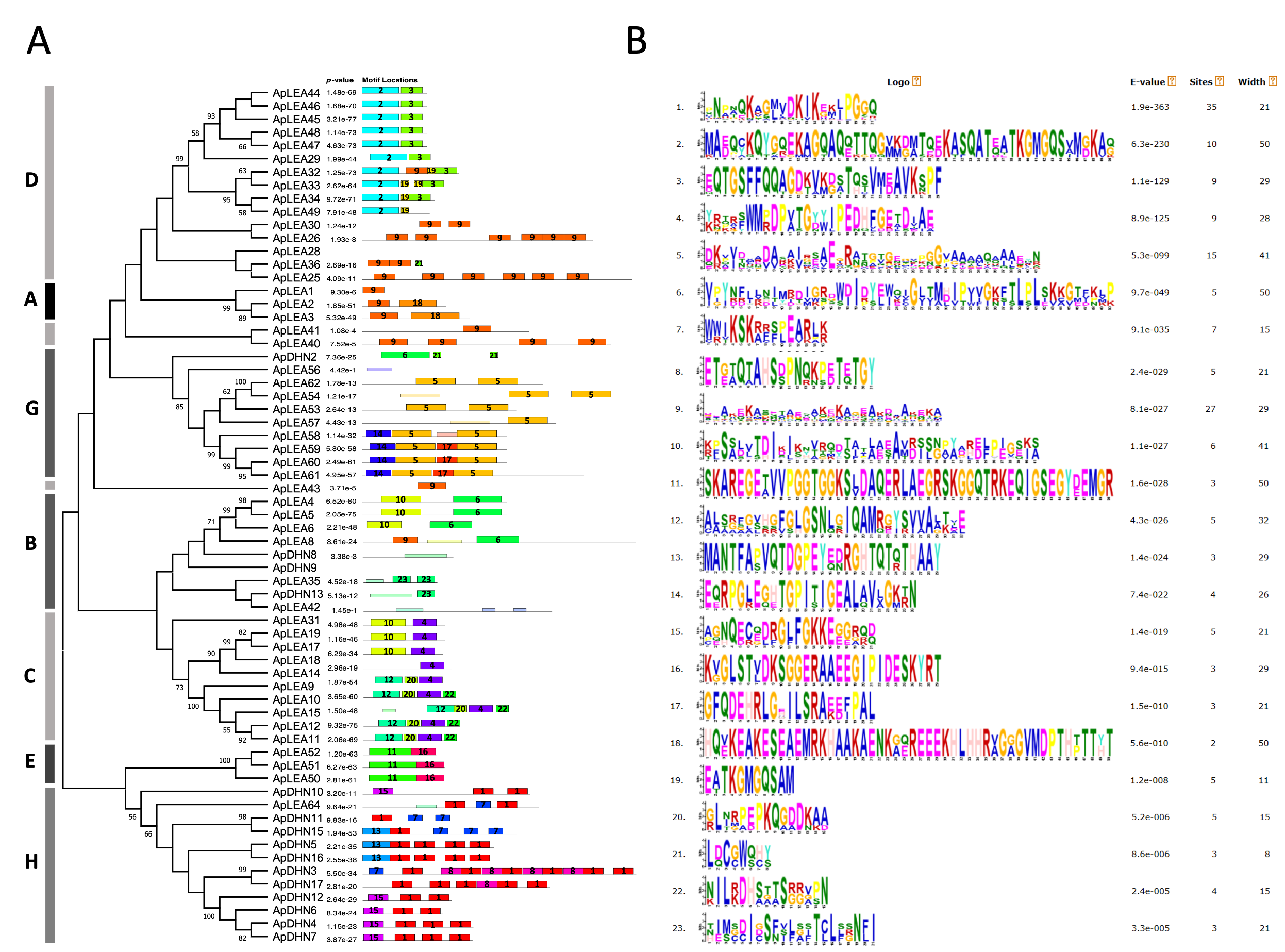

Supplement: Supplementary file 1 [file ijms-25-04820-s001.zip › Supplementary Figure S1. Phylogenetic relationships of A. pinsapo LEA sequences.png]

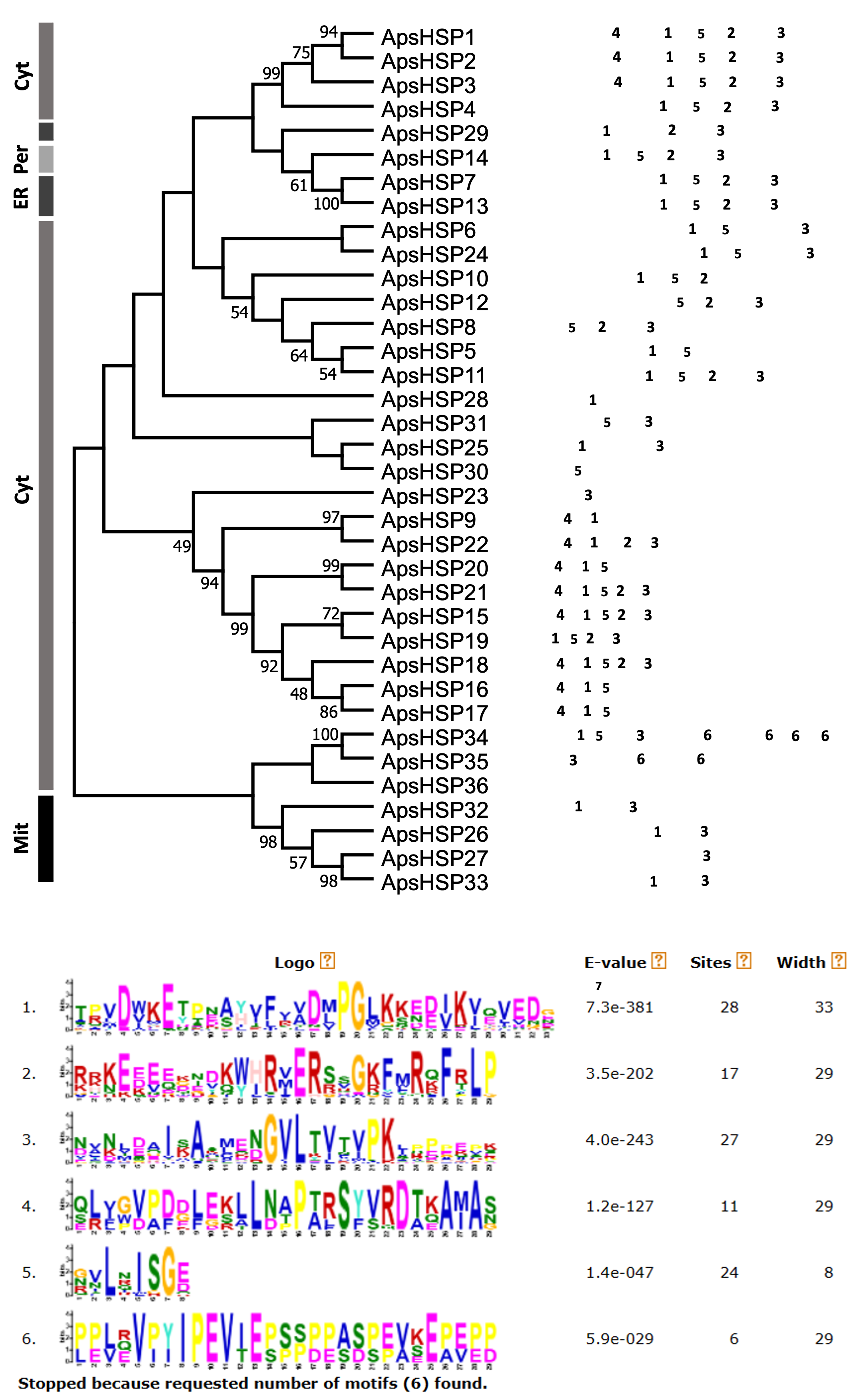

Supplement: Supplementary file 1 [file ijms-25-04820-s001.zip › Supplementary Figure S2. Phylogenetic relationships of A. pinsapo sHSP sequences.png]

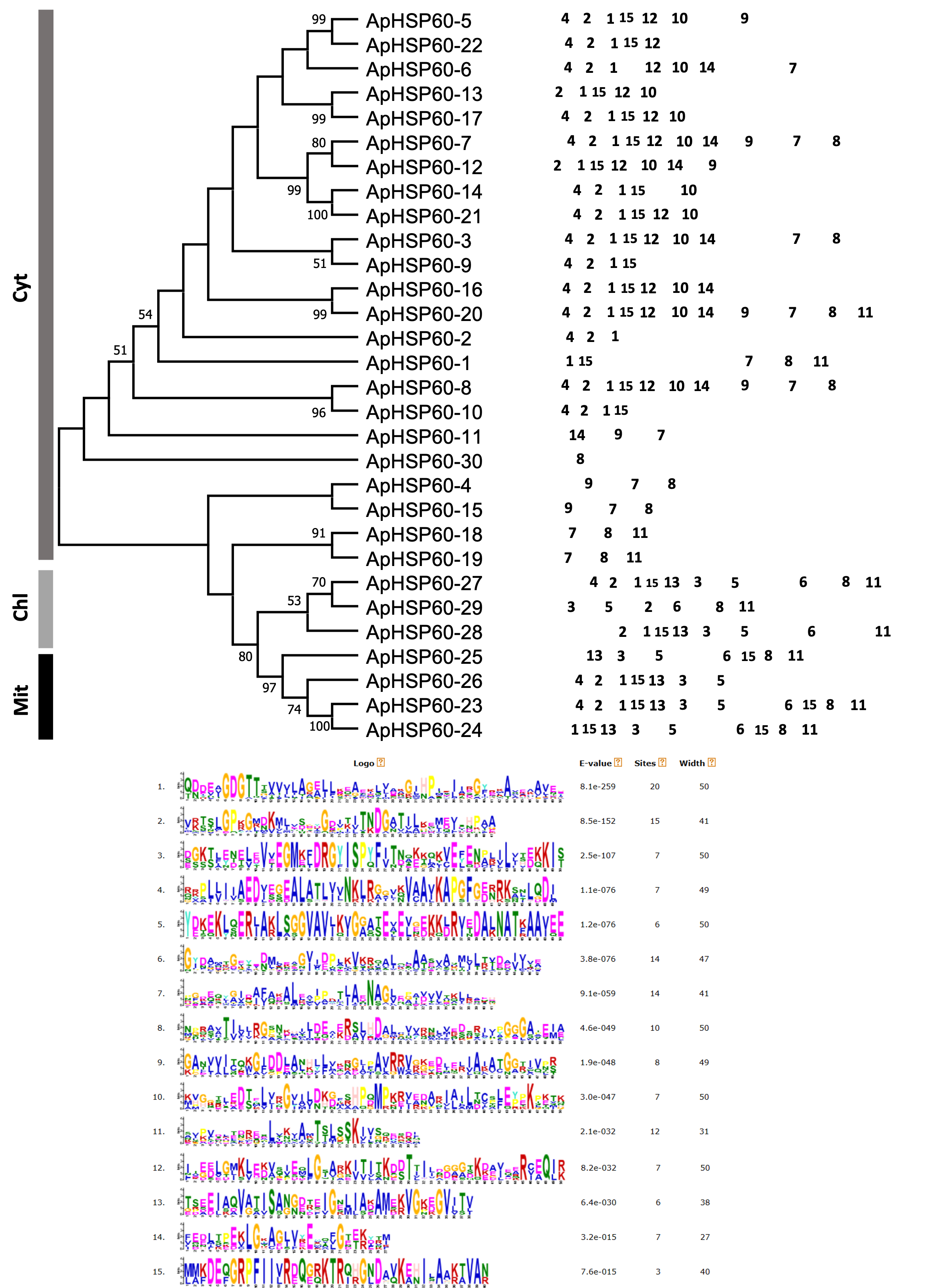

Supplement: Supplementary file 1 [file ijms-25-04820-s001.zip › Supplementary Figure S3. Phylogenetic relationships of A. pinsapo HSP60 sequences.png]

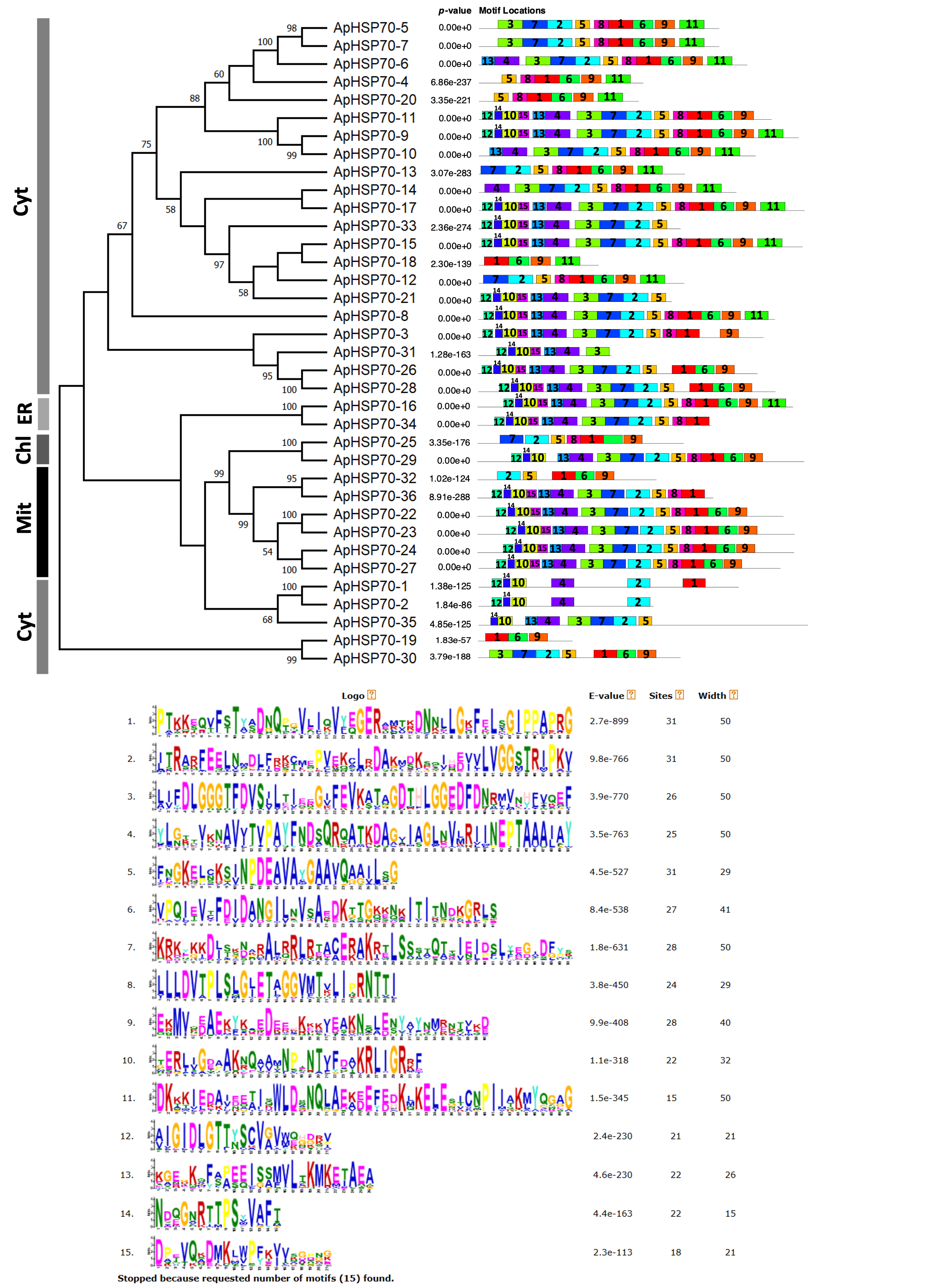

Supplement: Supplementary file 1 [file ijms-25-04820-s001.zip › Supplementary Figure S4. Phylogenetic relationships of A. pinsapo HSP70 sequences.png]

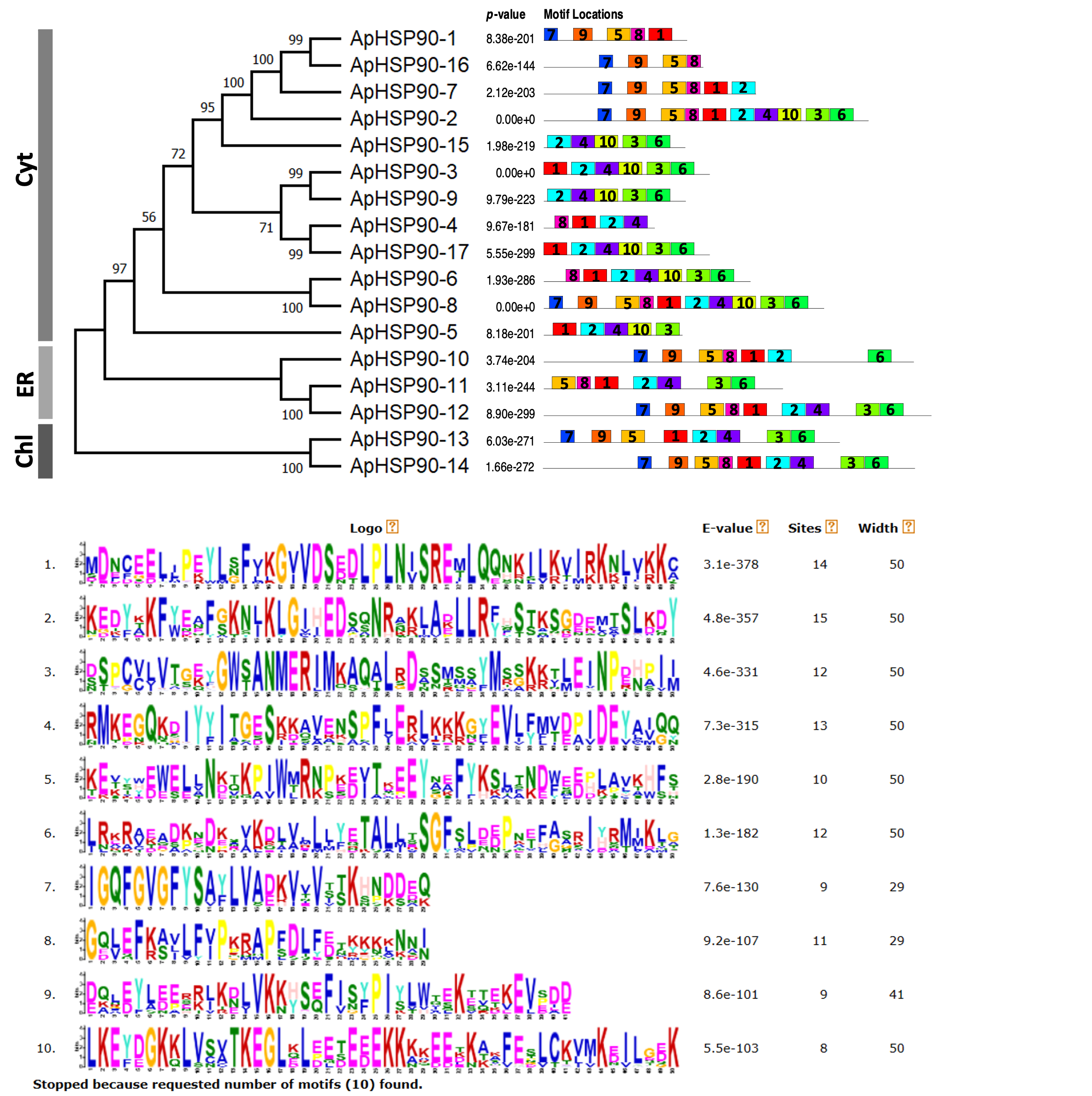

Supplement: Supplementary file 1 [file ijms-25-04820-s001.zip › Supplementary Figure S5. Phylogenetic relationships of A. pinsapo HSP90 sequences.png]

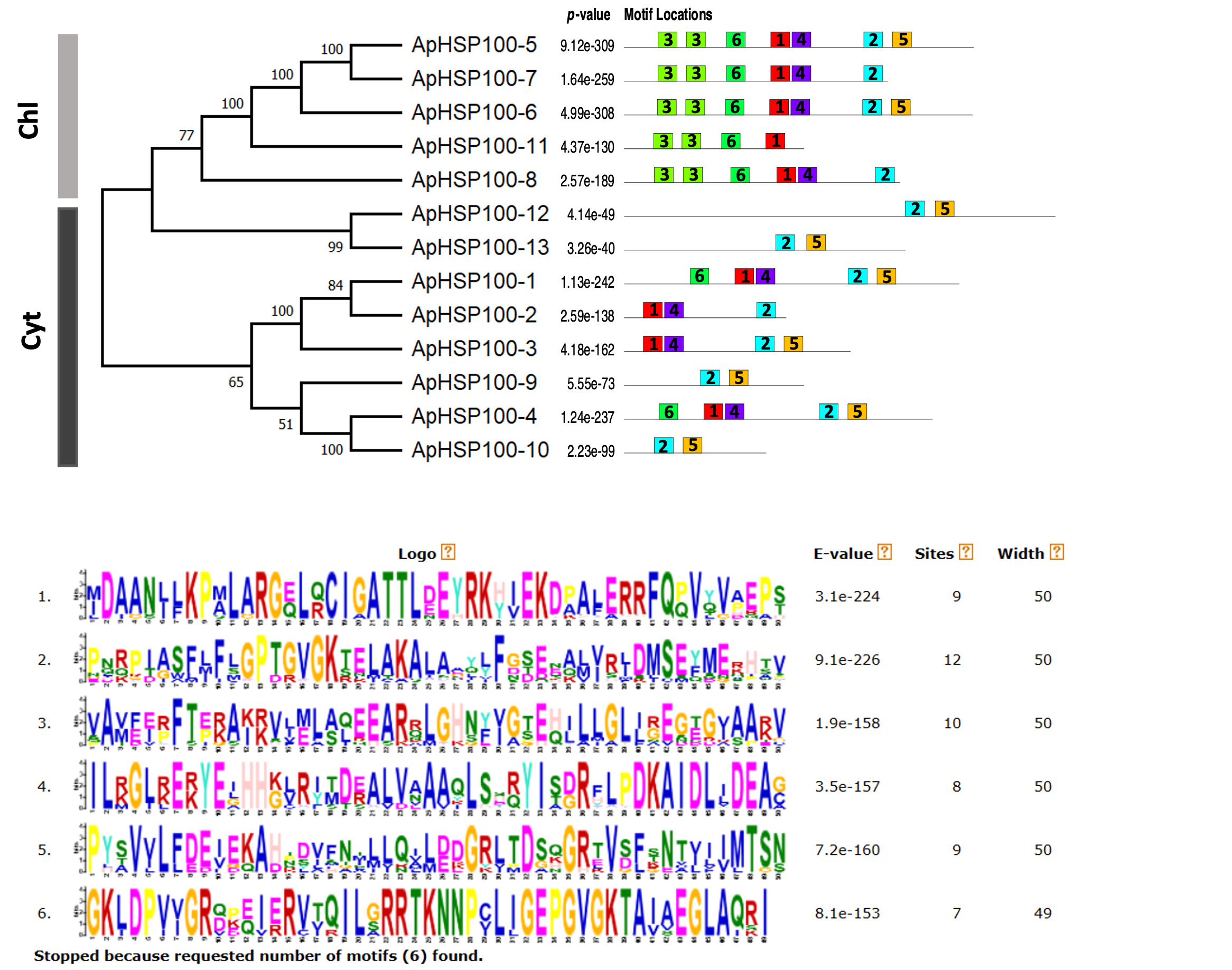

Supplement: Supplementary file 1 [file ijms-25-04820-s001.zip › Supplementary Figure S6. Phylogenetic relationships of A. pinsapo HSP100 sequences.png]

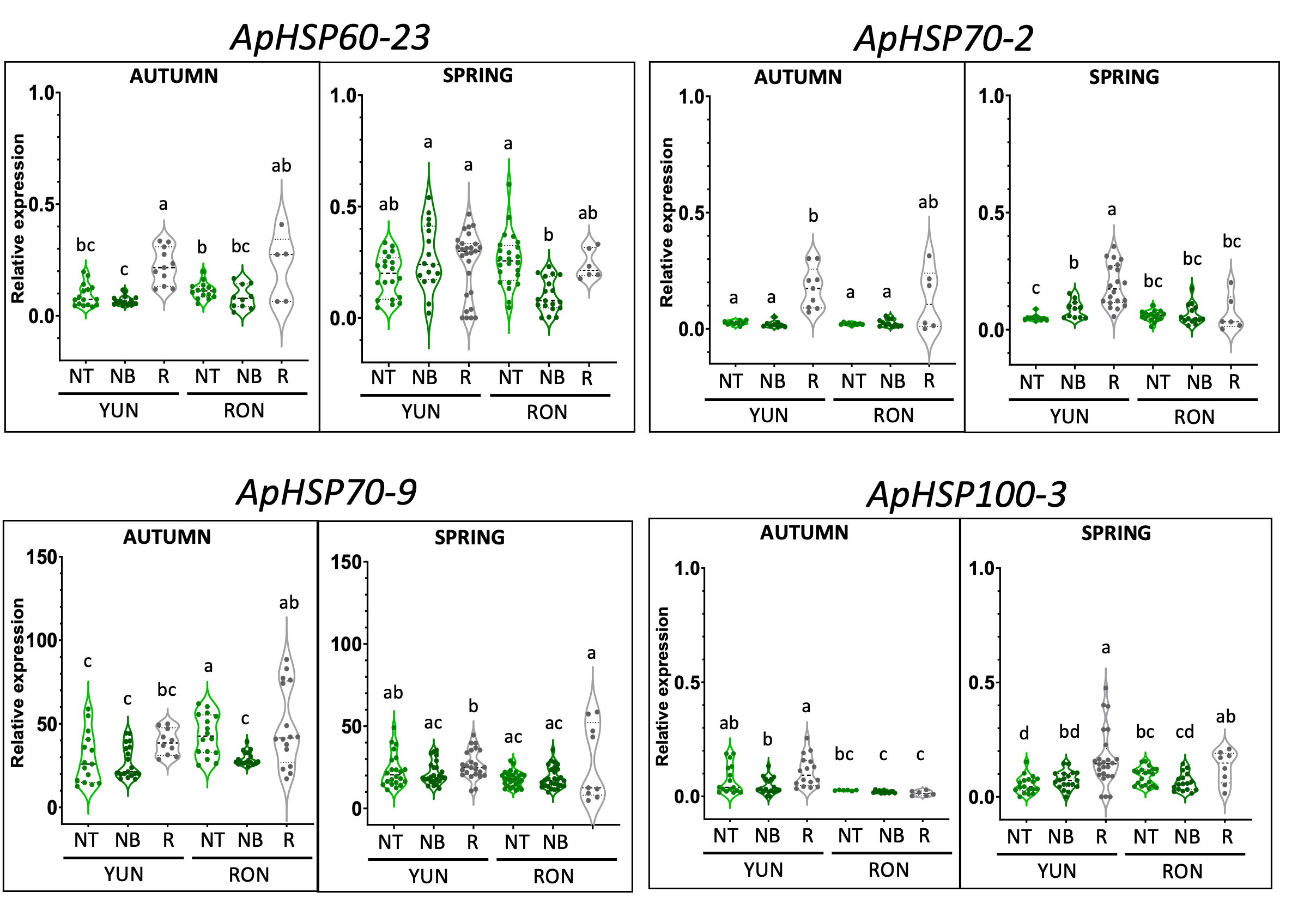

Supplement: Supplementary file 1 [file ijms-25-04820-s001.zip › Supplementary Figure S7. Expression profile of ApHSP60-23, ApHSP70-2,9, and ApHSP100-3.png]
